# Supplementary material for: Precise atom manipulation through deep reinforcement learning
Source: Nat Commun. 2022 Dec 5;13:7499. doi: 10.1038/s41467-022-35149-w (PMC9722711; doi:10.1038/s41467-022-35149-w)
Supplement: Supplementary file 1 — Supplementary Information [file 41467_2022_35149_MOESM1_ESM.pdf]

Supplementary information for "Precise atom manipulation through deep reinforcement learning"

I-Ju Chen,[*,†] Markus Aapro,[†] Abraham Kipnis,[†] Alexander Ilin,[‡] Peter Liljeroth,[*,†] and Adam S. Foster[*,†,¶]

†*Department of Applied Physics, Aalto University, Finland*

‡*Department of Computer Science, Aalto University, Finland*

¶*Nano Life Science Institute (WPI-NanoLSI), Kanazawa University, Kakuma-machi, Kanazawa 920-1192, Japan*

E-mail: i-ju.chen@aalto.fi; peter.liljeroth@aalto.fi; adam.foster@aalto.fi

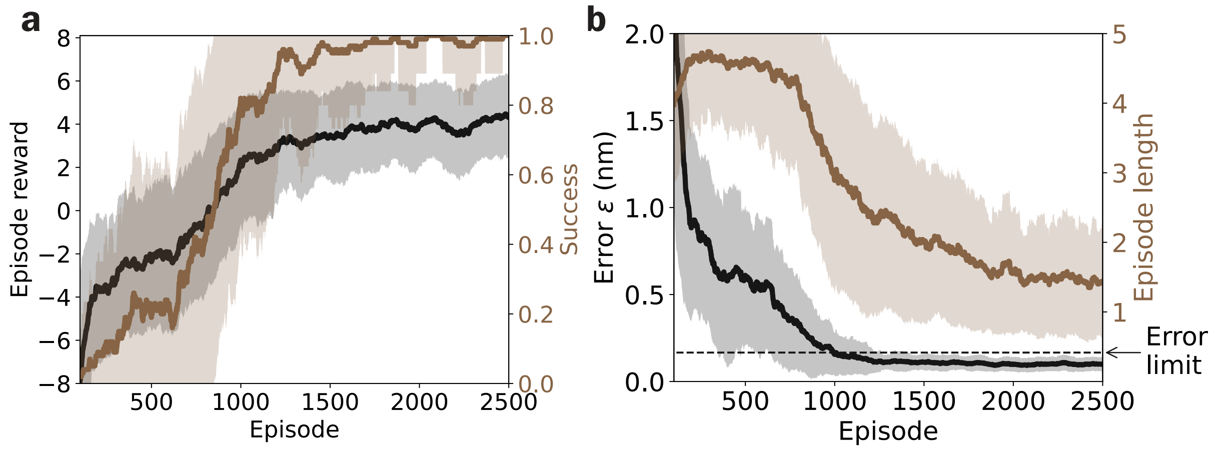

Supplementary Figure 1: **Co manipulation in the close proximity range** (a,b) The rolling mean (solid lines) and standard deviation (shaded areas) of episode reward, success rate, error, and episode length over 100 episodes showcase the training progress. The agent reaches optimal precision and 100 % success rate over 100 episodes after ∼ 2000 episodes, similar to the Ag agent shown in the main text.

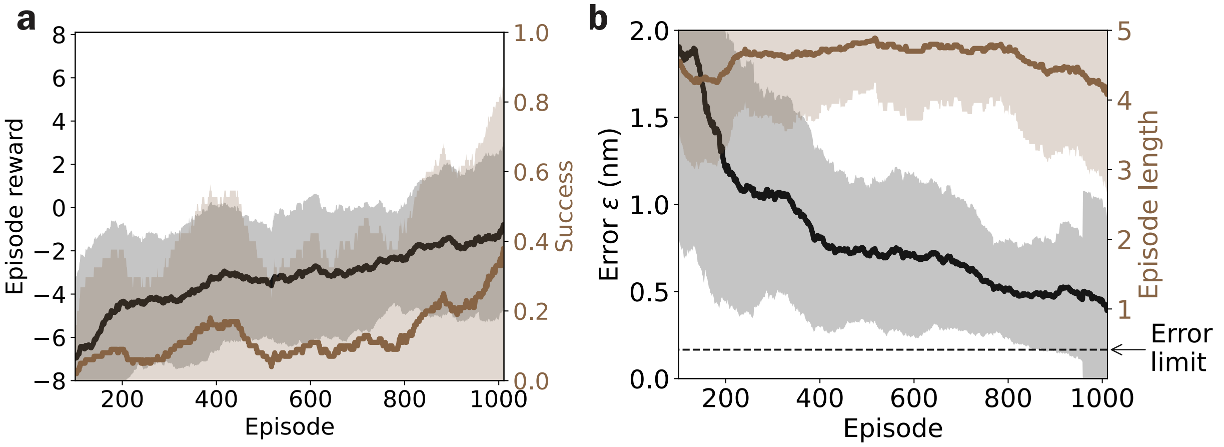

Supplementary Figure 2: **Co manipulation in the high bias range** (a,b) The rolling mean (solid lines) and standard deviation (shaded areas) of episode reward, success rate, error, and episode length over 100 episodes showcase the training progress. The training is terminated at the 1000th episode. Better manipulation precision and efficiency can be expected with more training.

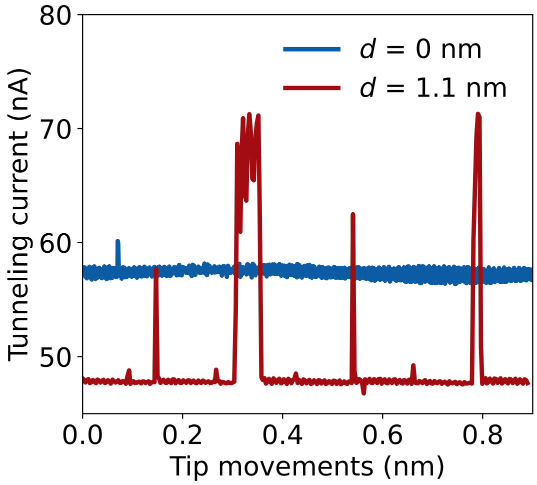

Supplementary Figure 3: **Tunneling current and atom movement** The two tunneling current traces obtained during manipulations show that they are highly indicative of atom movement distances d, which are extracted from STM scans.

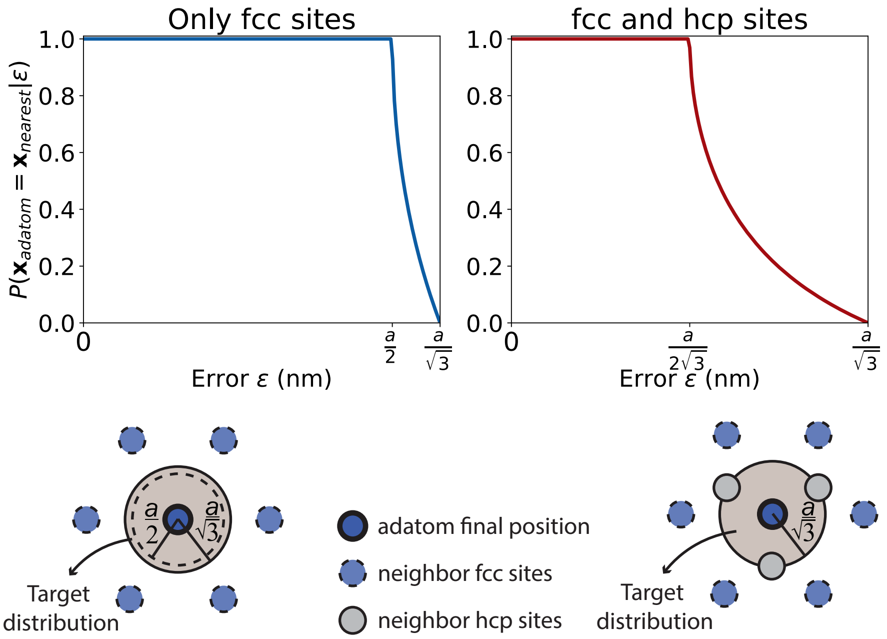

Supplementary Figure 4: **Probability of occupation and adsorption site geometry** The probability an atom is placed at the nearest site to the target at a given error $P(\mathbf{x}_{\mathrm{adatom}} = \mathbf{x}_{\mathrm{nearest}}|\varepsilon)$ is computed by integrating over possible target positions and considering two possible adsorption sites geometries: (1) only fcc sites can be occupied and (2) both fcc and hcp sites can be occupied.

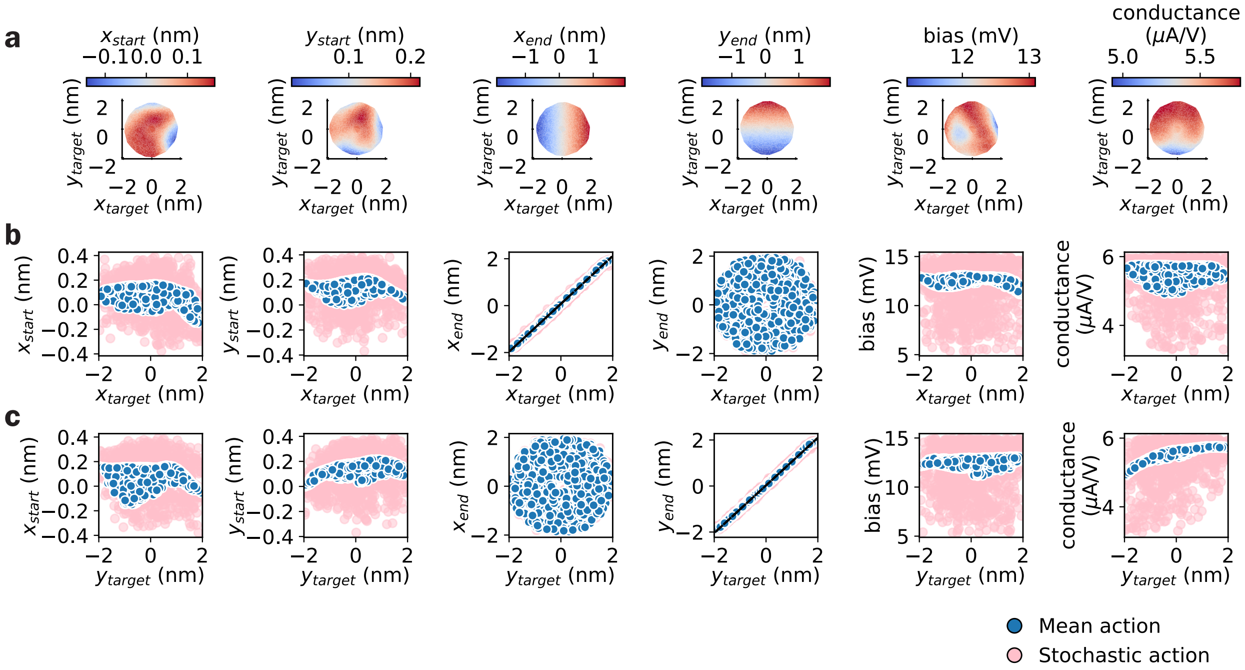

Supplementary Figure 5: **Action of a trained DRL agent** (a) Surface plot of the mean actions output by the trained DRL agent for 1000 randomly sampled states (x_{target}, y_{target}, $x_{\text{adatom}} = 0$, $y_{\text{adatom}} = 0$). (b, c) Scatter plot of the mean and the stochastic actions output by the trained DRL agent. The relation between x_{end} (y_{end}) and x_{target} (y_{target}) are well fitted by a linear model (black line).

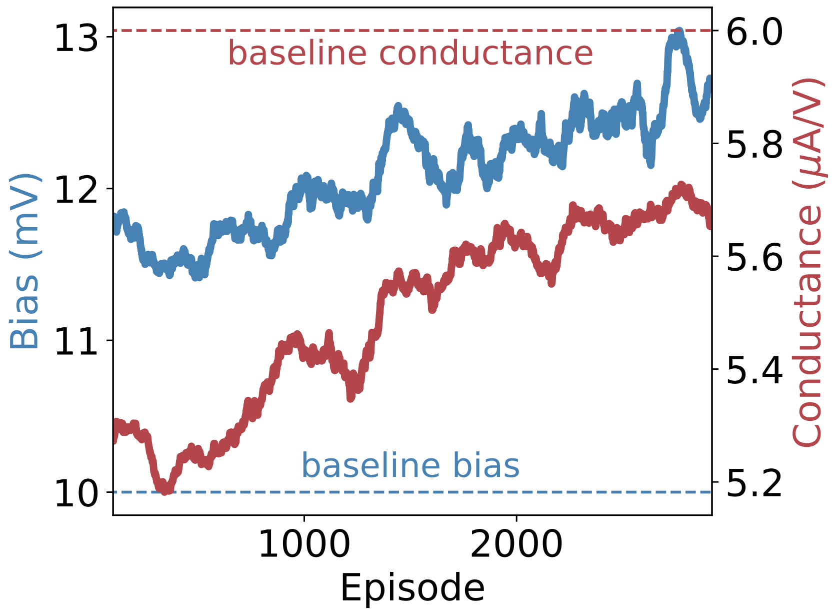

Supplementary Figure 6: **Average bias and conductance used by DRL agent** The rolling mean (over 100 episodes) of the biases and conductance used by the DRL agent in the training shown in Fig. 2. As the training progresses, the DRL agent applies larger bias and conductance to manipulate the atom.

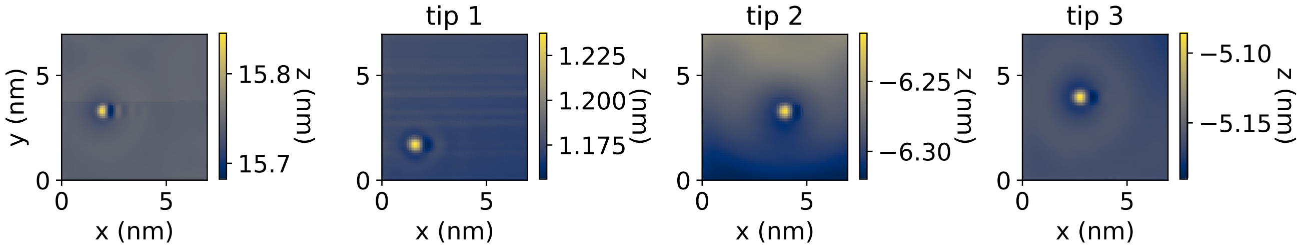

Supplementary Figure 7: **Effect of tip changes** The STM scans taken before and after tip changes led to tip conditions named tip 1, tip 2, and tip 3 as discussed in Fig. 2(a, b, d, e). Changes in tip heights and topographic contrasts can be observed.

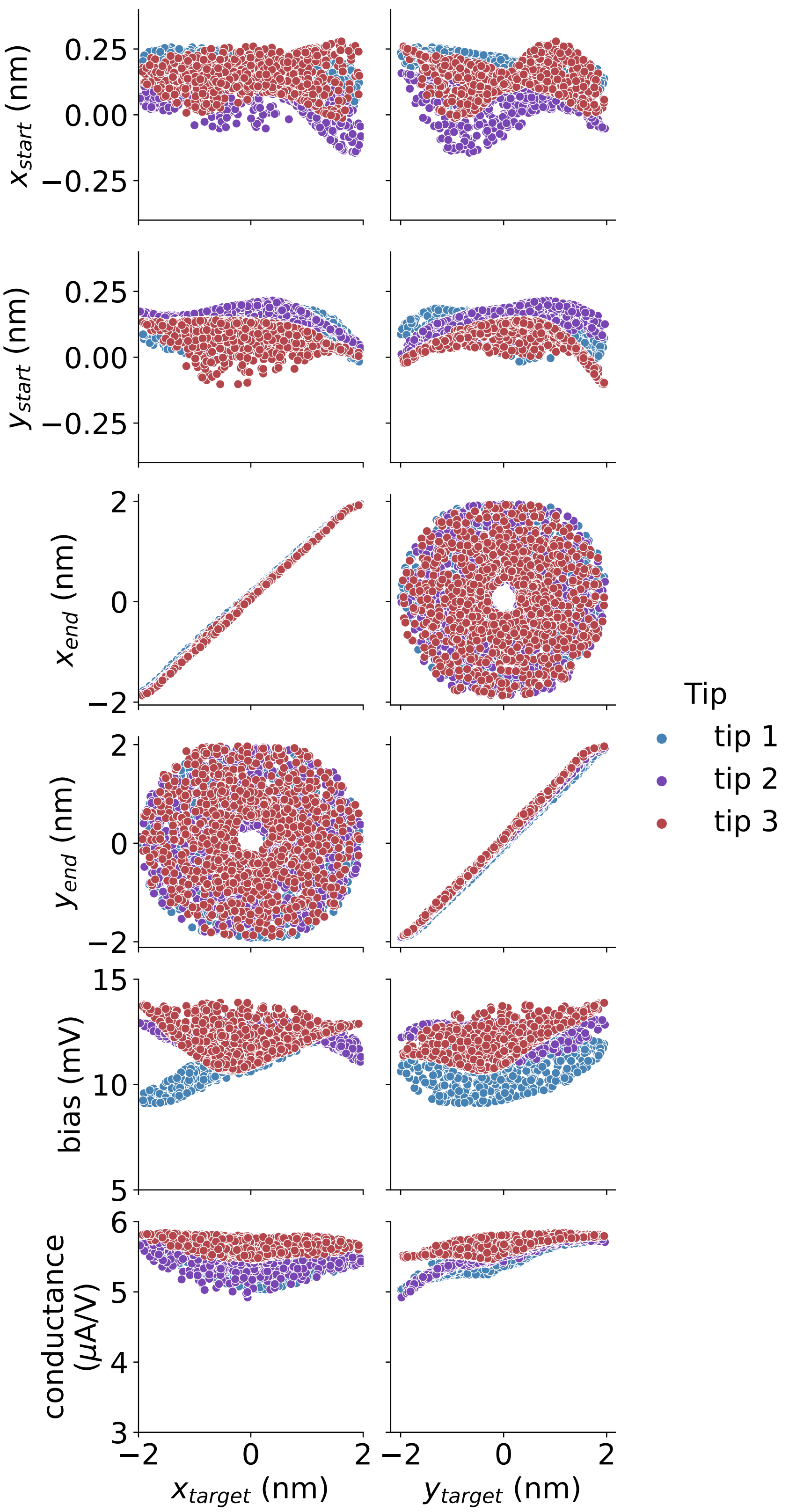

Supplementary Figure 8: **DRL actions under different tip conditions** The mean actions taken by the DRL agents after continued training under the conditions of tip 1, tip 2, and tip 3 for 1000 randomly sampled states (x_{target}, y_{target}, $x_{\text{adatom}} = 0$, $y_{\text{adatom}} = 0$).

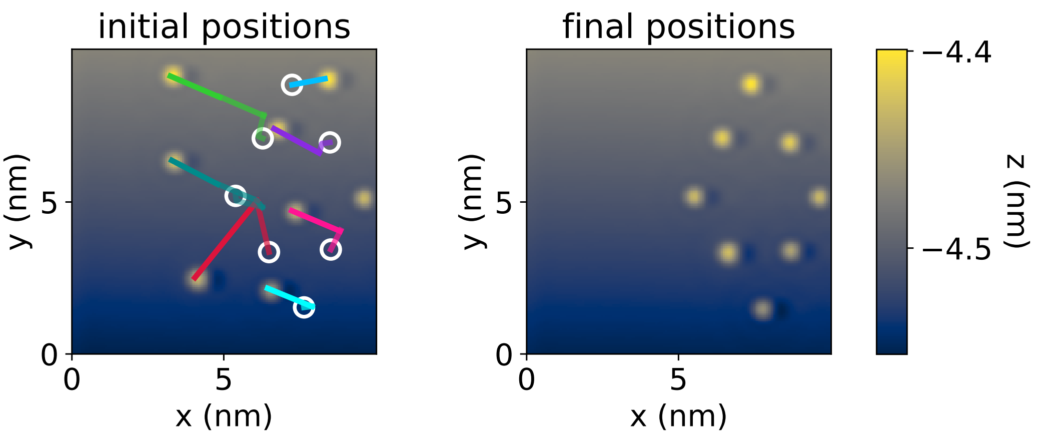

Supplementary Figure 9: **Building an 8-atom unit** The STM scans taken before and after building the 8-atom unit with the DRL agent. The colored lines indicate the manipulation paths and the white circles indicate the final positions of the adatoms.

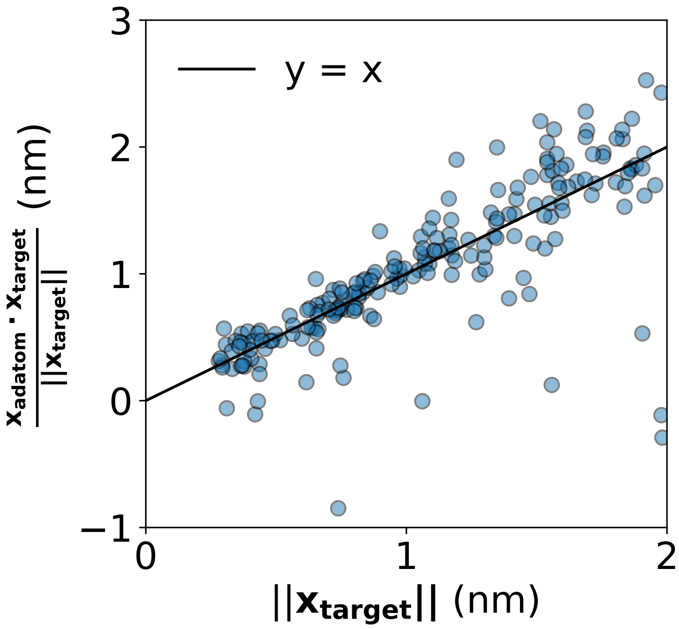

Supplementary Figure 10: **Performance of DRL agent trained with an alternative reward function** After training with a reward function including a term $r' \propto (\mathbf{x}_{\mathrm{adatom,t+1}} - \mathbf{x}_{\mathrm{adatom,t}}) \cdot \mathbf{x}_{\mathrm{target}}$ for 2000 episodes, the DRL agent shows a tendency to move the adatom overly far in the target direction.
